# Applying Acoustic Signals to Monitor Hybrid Electrical Discharge-Turning with Artificial Neural Networks

**DOI:** 10.3390/mi16030274

**Published:** 2025-02-27

**Authors:** Mehdi Soleymani, Mohammadjafar Hadad

**Affiliations:** 1 School of Mechanical Engineering, College of Engineering, University of Tehran, Tehran P.O. Box 14155-6619, Iran; soleimani.mehdi@ut.ac.ir; 2 Department of Mechanical Engineering, College of Engineering and Technology, University of Doha for Science and Technology, Doha P.O. Box 24449, Qatar

**Keywords:** hybrid processes, surface roughness, neural networks, multi-label classification, electrical discharge turning, deep learning

## Abstract

Artificial intelligence (AI) models have demonstrated their capabilities across various fields by performing tasks that are currently handled by humans. However, the training of these models faces several limitations, such as the need for sufficient data. This study proposes the use of acoustic signals as training data as this method offers a simpler way to obtain a large dataset compared to traditional approaches. Acoustic signals contain valuable information about the process behavior. We investigated the ability of extracting useful features from acoustic data expecting to predict labels separately by a multilabel classifier rather than as a multiclass classifier. This study focuses on electrical discharge turning (EDT) as a hybrid process of electrical discharge machining (EDM) and turning, an intricate process with multiple influencing parameters. The sounds generated during EDT were recorded and used as training data. The sounds underwent preprocessing to examine the effects of the parameters used for feature extraction prior to feeding the data into the ANN model. The parameters investigated included sample rate, length of the FFT window, hop length, and the number of mel-frequency cepstral coefficients (MFCC). The study aimed to determine the optimal preprocessing parameters considering the highest precision, recall, and F1 scores. The results revealed that instead of using the default set values in the python packages, it is necessary to investigate the preprocessing parameters to find the optimal values for the maximum classification performance. The promising results of the multi-label classification model depicted that it is possible to detect various aspects of a process simultaneously receiving single data, which is very beneficial in monitoring. The results also indicated that the highest prediction scores could be achieved by setting the sample rate, length of the FFT window, hop length, and number of MFCC to 4500 Hz, 1024, 256, and 80, respectively.

## 1. Introduction

AI models have played a significant role in enhancing the performance of various processes, including those in manufacturing [1]. AI models can help reduce manufacturing costs while enhancing process stability and product quality [2]. Support vector machines, artificial neural networks, adaptive neuro-fuzzy, and regression models can classify weld defects or predict the strength of welded joints by training with radiographic images, surface images, or other welding features [3,4,5]. Artificial Neural Networks have also demonstrated a strong performance in predicting beam quality [6], failure load, over-weld, normal weld, and under-weld conditions [7], as well as the static and fatigue behavior of joints [8]. To address complex prediction problems with mathematical limitations, AI can be an effective solution [9]. AI models have been widely employed to assess mechanical properties such as hardness [10,11,12,13], elastic limit [10], yield strength [10], and residual stress [13,14,15]. Although most of the applied AI models utilize numerical, categorical, or image-based data for their input training, some researchers have opted for acoustic data and applied their trained models across various industries. For example, breathing sounds were used to train an AI model to predict COVID-19 [16]. Additionally, acoustic signals generated during the movement of a car wheel on the road were used to classify the wetness of road surfaces [17]. AI models can make it easier to improve vehicle safety by classifying road surface roughness [18], monitor roads by classifying asphalt damages [19], and monitor pavement quality [20], all by training AI models with acoustic signals.

**Figure 1:**
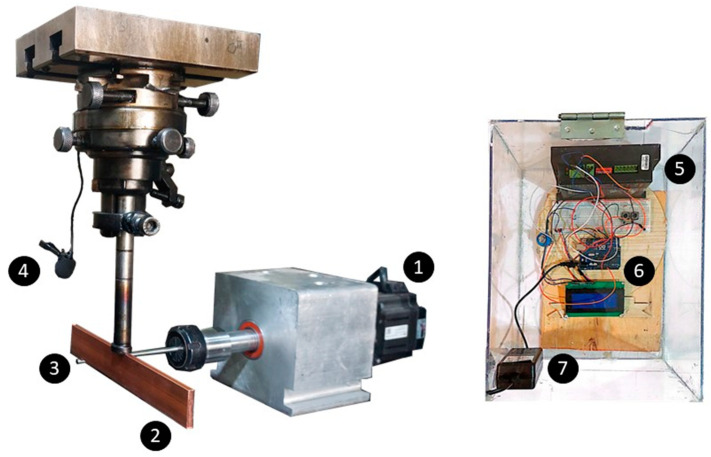
EDT setup, the driver and controller, and the recording device applied for machining and signal recording.

Acoustic data are also valuable for training AI models used in manufacturing processes. For example, welding defects can be detected using cross-validation and support vector machines in Gas Tungsten Arc Welding (GTAW) [21]. In another study, the sound of arcs generated during MIG/MAG was utilized to train an Artificial Neural Network (ANN) model to monitor the quality and stability of the process [22]. Also, in order to automate the GTAW process and control welding penetration, the training features were extracted from acoustic signals of GTAW where an ANN model was trained as the predictor model [23]. Acoustic signals could be used for training monitoring models in other manufacturing processes. An AI model was trained to monitor the tool wear in milling operations [24] and an accurate ANN model was trained to predict the degree of tool wear in milling processes [25]. The researchers mentioned that they were able to develop a tool monitoring model with a simple structure when they used acoustic signals as the training data [26]. The depth of cut, feed rate, and rotational speed were predicted by a deep neural network model trained by the acoustic emission signals in the turning process [2]. When turning Inconel 718, the resulting surface roughness was predicted by training a convolutional neural network using signals acquired from acoustic emission [27]. Multi-Layer Perceptrons, CNNs, Long Short-Term Memory (LSTM) networks, and transformer models were trained using sound and force data generated during the machining process to classify surface roughness [28]. Additionally, feeding a convolutional neural network with sounds recorded during the grinding process enabled the intelligent model to predict conditions of the grinding wheel [29]. Tool wear was evaluated by examining the tool flank during the turning process using conventional methods, including Logistic Regression and Neural Networks. By utilizing the sounds generated during the turning process, the researchers were able to classify the condition of tool wear [30]. A similar study was conducted to monitor tool wear values by preprocessing the machining audio signals by Fast Fourier Transform and training them with a convolutional neural network model [31]. Figure 1 shows the main components of the electrical discharge turning (EDT) process, the rotating workpiece and the tool electrode. EDT is a form of electrical discharge machining (EDM) which benefits from both EMD and turning processes in machining the difficult-to-machine bars as a hybrid process [32]. Pulse-on time and peak current effect the surface roughness when machining the Ti-6Al-4V by EDT [33,34]. Among the discharge current, magnetic field, pulse on time, and rotational speed, the magnetic field had the most significant effect on the EDT results [35]. The evaluation of the effects of workpiece rotational speed, electrode shape, and jump down time on circularity deviation and surface roughness revealed that workpiece rotational speed and electrode shape had the most significant impacts on surface roughness in the EDT process. In fact, increasing the rotational speed leads to a better flushing of debris, resulting in a smoother surface [36]. The effect of rotational speed, pulse on-time, and discharge current on MRR, surface roughness, and tool wear was studied on AISI L2 steel in the EDT process [37]. Increasing the rotational speed resulted in better flushing and an increased MRR. Additionally, higher rotational speeds improved surface roughness by reducing the recast layer and enhancing flushing [38]. When machining AISI D2 steel by incorporating a magnetic field into the EDT, the magnetic flux and rotational speed were reported as the most important parameters that affect surface integrity [39]. The optimization of accuracy and precision in the (EDT) process using Taguchi robust design revealed that the most important parameters affecting MRR are pulse-on time, servo control, intensity, and rotational speed [40].

In training a conventional classification model, the model should be able to detect the only output label, which is related to the input data. But, in training a multilabel classification model, the trained model should learn how to predict the multiple output classes for a single input. For example, when predicting the gene categories in biology, the gene can be labeled as its metabolism, protein synthesis, and transcription simultaneously. If the L = { $\lambda_{1},\;\lambda_{2},\;\lambda_{3},\;\ldots \;\lambda_{m}$ } is considered the class labels and X as an instance space in a multilabel model, any instance like x ∈ X can be related to a subspace of labels L ∈ $2^{L}$, which are the subset of relevant labels, and L\L is its complement including the irrelevant labels. After training, the multilabel classifier should be able to classify h: X → $2^{L}$ when T is a finite set of recorded data as training data which is in the form of tuples (x, $L_{x}$) ∈ X × $2^{L}$. The prediction is being performed according to the specified loss function and minimizing loss function [41].

Although there is a considerable amount of research on evaluating the ability of AI models to enhance the prediction and monitoring of manufacturing processes, researchers often struggle to provide sufficient data for training such models, which is crucial for developing an accurate and reliable model. Since preparing large datasets and numerous images as typical training data is very time-consuming and costly, we decided to extract the training data from the sounds generated during the processes. The approach of using signals like the acoustic emission signals was used by the previous researchers to detect the depth of cut, feed rate, and spindle speed in the turning process [2] or to predict the surface roughness [27], but the acoustic emission method is globally a high-cost method and needs to multiple especial sensors. So, we proposed using a microphone to record the audio-based signals as the training data which is a simple and low-cost method. Additionally, the sounds produced by the devices provide significant information that can be easily recorded with a microphone [42]. As some researchers [40] have pointed out, the hybrid EDT process presents various parameters that need optimization; thus, we selected this process as the focus of our evaluation. The input process parameters included workpiece rotational speed, pulse on-time, and discharge current, which many researchers have identified as significant parameters affecting EDT outputs. We could have trained different AI models to predict only one factor, such as the surface roughness, using the input acoustic signals with typical multiclass classification models. In such a case, multiple models to predict each special output would have to be trained. Instead, we aimed to improve the training and predicting tasks by developing a multilabel classification model, which is rarely seen in similar manufacturing applications. This way, we could train one model which is able to predict multiple parameters simultaneously. This AI multilabel classification model is expected not only to predict the surface roughness based on the given acoustic signals, but also to detect the values of workpiece rotational speed, pulse on-time, and discharge current corresponding to the recorded acoustic signals. While predicting the surface roughness could be an alternative method to evaluate the machined surface condition, predicting the value of the mentioned input factors could help to monitor the EDT condition by a new online method using a simple microphone. The model, which could have been trained on fewer prediction labels in a standard classification model, should learn to differentiate among numerous labels in a multilabel classification setting when given a recorded acoustic signal.

## 2. Material and Methods

### 2.1. Experimental Procedure

To conduct the EDT process as a hybrid process of Turning and EDM, a rotating mechanism was mounted on an EDM machine, as shown in Figure 1. The stepper motor driver (depicted as 5) and the connected microcontroller (depicted as 6) precisely supply the required power to the stepper motor (depicted as 1) and control the set value of the rotational speed. The number 7 shows the adapter for the microcontroller. A high-speed steel bar (depicted as 3) with a 5 mm diameter was used as the workpiece. Given that natural erosion occurs either in the workpiece or the tool electrode during the EDT process, the copper strip used as the tool electrode (depicted as 2) and the workpiece were long enough to be repositioned after each test. This ensured that all tests experienced similar conditions under constant parameters. The microphone (depicted as 4), used to record sounds during the process, was a COMICA SIG.LAV V05 and was positioned near the machining area, as shown in Figure 1. Its specifications are demonstrated in Table 1.

**Figure 2:**
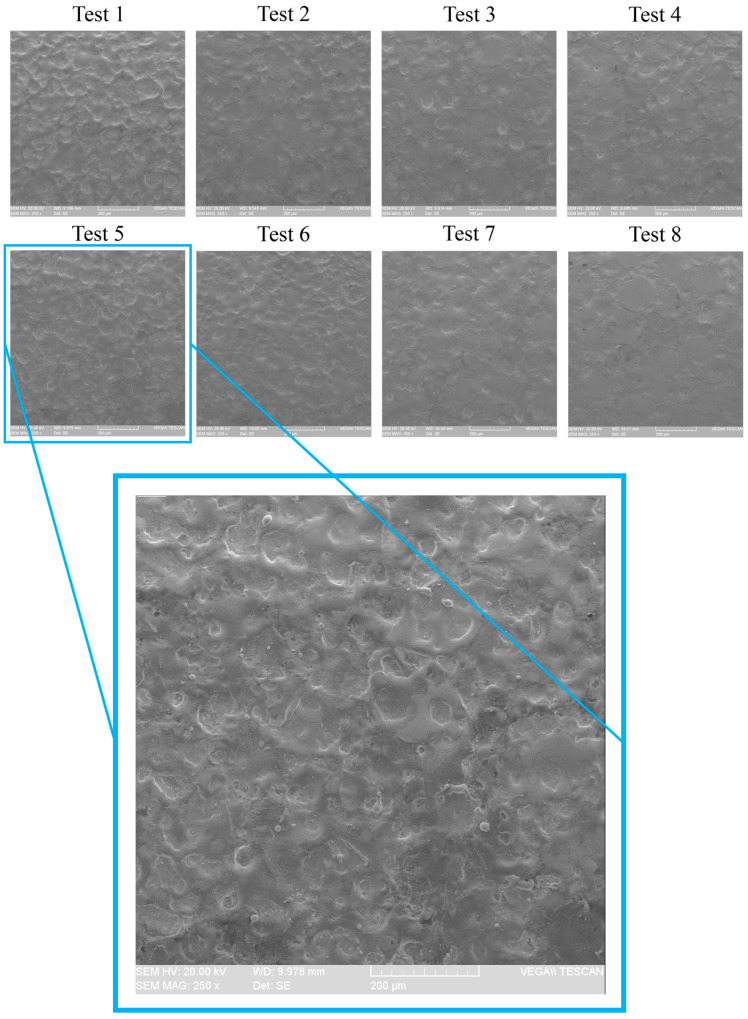
Scanning Electron Microscope images from the machined surface.

In order to record the sound from the machining area, two different values were considered for each input parameter, specifically the workpiece rotational speed (10–20 rpm), pulse on-time (100–200 µs), and discharge current (1.5–2.5 A). All possible combinations of these parameters were evaluated, leading to eight different tests conducted in the EDT process, as shown in Table 2. During each machining test, the generated sounds were recorded, and the recorded files were split into shorter segments to prepare the data for preprocessing and feeding into the AI model. In total, out of the 3289 split acoustic files, 2631 files were designated as training and validation data for model training, while 658 files were reserved as test data, which were not included in the training set for the final evaluation of the trained model. Additionally, the surface roughness resulting from each experiment was measured using a HOMMELWERKE Turbo Roughness Tester (V3.34), and the value recorded for each test was used as the surface roughness for each trial, as depicted in Table 2. Additionally, Figure 2 shows the surface of the workpiece machined by the EDT process which underwent surface roughness testing.

### 2.2. Input Preprocessing

To make analog acoustic signals interpretable for the AI model, they must be preprocessed and converted into digital signals. Furthermore, the models need to receive data extracted from these signals as training features, so it is essential to use a feature extraction method.

One of the methods for extracting acoustic features is applying the MFCC algorithm, which involves a short-term analysis of the acoustic signals [43]. First, the acoustic signals are transformed using the Fourier transform. Next, triangular windows are applied to map the spectrum powers onto the Mel scale. After this, the logarithm of the powers is calculated and transformed using the discrete cosine transform, and the Mel frequencies are also computed. Finally, the feature vectors are obtained as the amplitudes of the spectrums [44].

**Figure 3:**
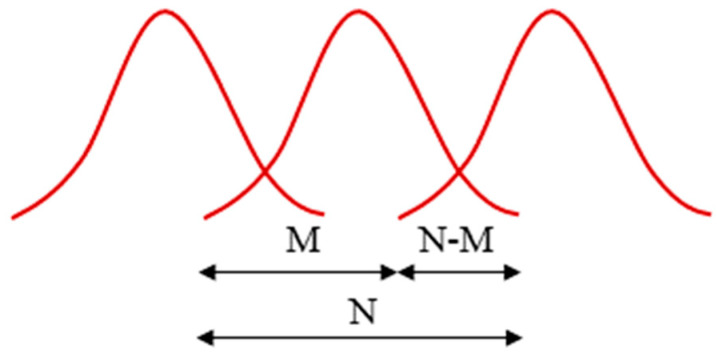
Schematic of windows and the segmenting of frames.

The first step in extracting MFCC involves segmenting the acoustic signals into overlapping frames, as illustrated in Figure 3. Here, N denotes the length of each frame, and each frame overlaps with its neighboring frames by a length of N-M. By applying a window function, such as the Hamm or Hann window (as shown in Equations (1) and (2)), to the segmented and overlapped frames, the discontinuities in the resulting signal frames are minimized. Consequently, discrete windows are produced in the time domain, which can subsequently be transformed into the frequency domain using the Fourier transform, resulting in *S*(*k*), which is the result of the frame with N samples [45].

(1) $$\left(\mathrm{Hamming}\;\mathrm{Window}\right)\;w\left(n\right)=0.54-0.46cos(\frac{2\pi n}{N-1}),\;0\leq n\leq N-1$$

(2) $$\left(\mathrm{Hanning}\;\mathrm{Window}\right)\;w\left(n\right)=0.5-0.5cos(\frac{2\pi n}{N-1}),\;0\leq n\leq N-1$$

Mel Filter Banks convert the resulting spectra to the Mel scale. In fact, the linear scale frequencies (in Hz) will be mapped in the Mel scale by the Mel Bank which includes the triangular-shaped Mel scale filters. Equation (6) is the formula which gives Mel frequency (Mel(f)) when ‘f’ is the linear frequency (in Hz). Also, $S_{mel}$ illustrated in Equation (7) is the result of multiplying the filter banks matrix (W) by the power spectrum (P). The log of the Mel scale spectrum is transformed by the discrete cosine transform, resulting in MFCCs without complex numbers. The discrete cosine transforms also make it easier to train the AI models by creating uncorrelated features. These final procedures have been depicted in Equations (8) and (9). If g = 0, 1, 2, …, J-1 (J is the number of MFCC), the $C_{g}$ will be the $g^{th}$ vector of MFCC and $S_{Mel}(m)$ will be the $m^{th}$ Mel filter if the number of Mel filters in the filter bank is $N_{f}$. Usually, many MFCC are not needed; the first few coefficients are sufficient to represent the input signal [45].

(3) $$S\left(k\right)=\sum_{n=0}^{N-1}s(n)e^{-j2\pi nk/N},\;0\leq n\leq N-1$$

(4) $$N_{2}=\frac{N}{2}$$

(5) $$P={\left|S(1:N_{2})\right|}^{2}$$

(6) $$Mel\left(f\right)=2595\times {log}_{10}\left(1+\frac{f}{700}\right)$$

(7) $$S_{mel}=W\times P$$

(8) $$C_{g}=dct({log}_{10}(S_{Mel}))$$

(9) $$C_{g}=\sqrt{\frac{2}{N_{f}}}\sum_{m=0}^{N_{f}-1}{log}_{10}(S_{Mel}(m))cos\left[\frac{g\pi }{N_{f}}(m+0.5)\right]$$

In the current investigation, the Librosa package [46] was used as the feature extraction tool to process the input acoustic signal and extract the MFCC. Although this package can process the input signal and extract MFCC by default, a closer examination reveals that there are several adjustable processing parameters. These include the number of MFCC (n_mfcc), the sample rate (sr), the number of samples between successive frames (hop_length), and the length of the FFT window (n_fft). Therefore, we have decided to optimize these parameters to ensure the highest classification scores. Overall, the values of current, Ton, rotational speed, and surface roughness were labeled from zero to thirteen and the aim was to detect and predict the value corresponding to them separately based on the given acoustic signal to the multi-label ANN model.

## 3. Results and Discussion

**Figure 4:**
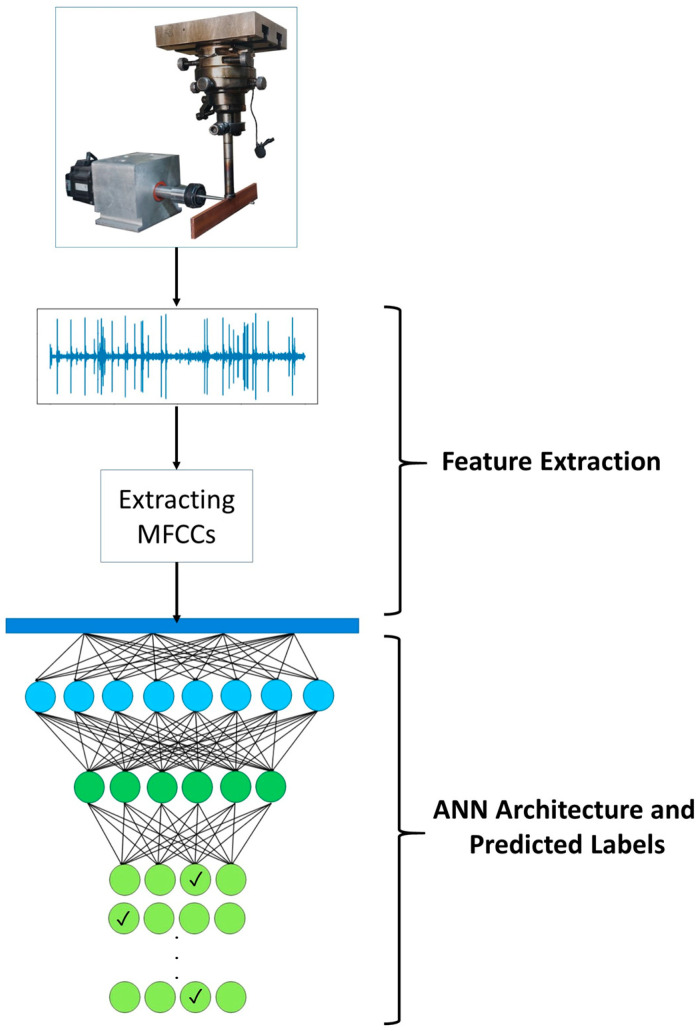
Schematic of feature extraction and the ANN architecture in which each hidden layer has a different color and each tick mark is the representative of the selected label.

Given the numerous hyperparameters in an ANN model, there are many possible structures for training. Finding the optimal combination of these parameters often requires extensive trial-and-error. Methods such as Keras Tuner [47] can help us address this challenge. The tuner can identify the best combination of model hyperparameters based on the ranges outlined in Table 3. Figure 4 illustrates the overall trend of feature extraction and the ANN structure.

**Figure 5:**
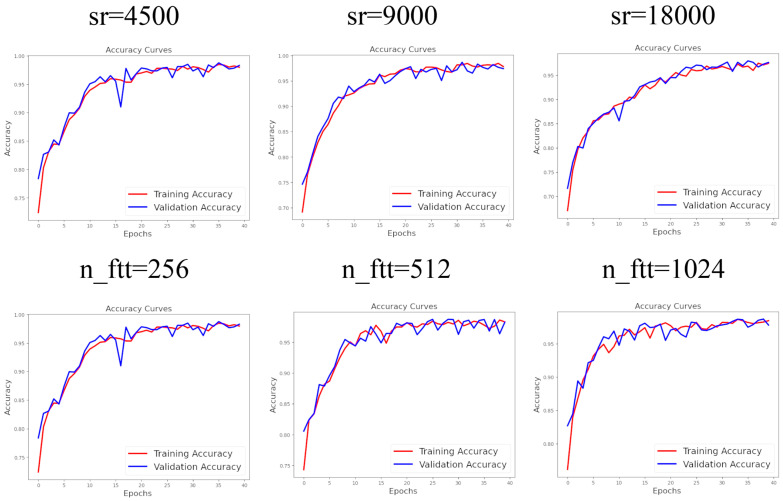
Accuracy diagrams for different sample rates (sr) and numbers of fft (n_fft).

**Figure 6:**
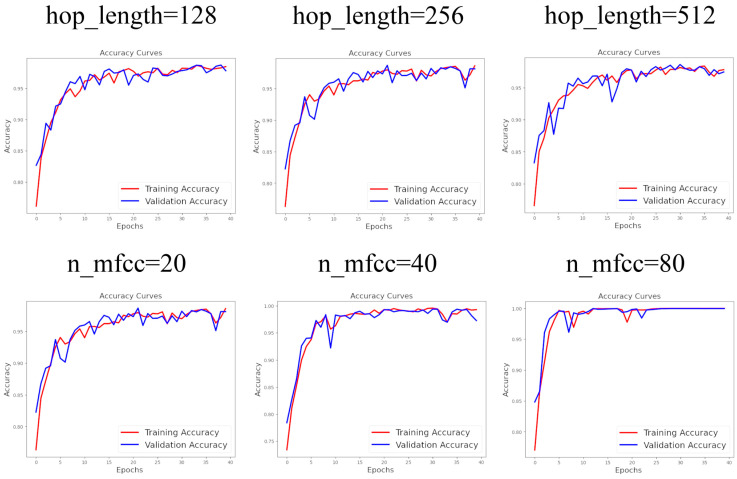
Accuracy diagrams for different numbers of MFCC (n_mfcc) and hop length.

As discussed, achieving optimal classification performance requires determining the best hyperparameters. To find the most effective preprocessing hyperparameters, we altered them systematically, and, ultimately, the highest predictive scores indicated the ideal hyperparameters. The sampling rate is one such hyperparameter that defines the frequency of extracting discrete values from a continuous signal. In the first step, the best model with the optimal sampling rate was selected and used to identify the best model with the most effective n_fft in the subsequent step. The researchers used a special value of preprocessing factors. For example, a 22,100 Hz or 20,000 Hz sample rate were applied in audio classifying [48] while another classifier used 22,050 Hz as its sample rate alongside the number of FFT of 2048 and a 512 hop length [49,50]. Also, the researchers usually use a number of MFCC between 10 and 20 [51]; however, 40 MFCC have also been used [52]. The value of the preprocessing factors highly influences the extracted information and, as such, the final trained model’s performance. So, in order to find probable better preprocessing values producing a more accurate trained classifier, we examined other preprocessing values which were not very far from the values studied by the previous researchers but were not exactly the same. Our process allowed us to systematically optimize hyperparameters, including sr (among 4500, 9000, and 18,000 Hz), n_fft (among 256, 512, and 1024), hop_length (among 128, 256, and 512), and n_mfcc (among 20, 40, and 80) in four distinct steps. To evaluate the performance of each model, three significant criteria were considered: precision, recall, and F1 score. Precision is calculated by dividing the number of test samples that have been correctly predicted as positive by the total number of test samples predicted as positive (both true positives and false positives). Recall, on the other hand, measures the fraction of test samples that have been correctly predicted as positive out of the total number of actual positive samples. Both precision and recall values indicate the improved performance of our AI model when they have higher values; however, the highest possible value for these scores is 1. Additionally, the F1 score is defined as the weighted harmonic mean of precision and recall, providing an overall depiction of the model’s performance [53]. As mentioned earlier, all the data (the features extracted from the acoustic signals) is divided into training, validation, and test sets. The model learns to recognize the true labels when it encounters any of the training data. Meanwhile, it evaluates its performance by testing its predictions on the validation data. If the model does not perform well on the validation data, it will adjust its predictions to improve. Typically, the loss should decrease while the accuracy should increase during the training process. Although achieving high accuracy on both the training and validation datasets suggests that the model has been well trained, our experience shows that even if both the training and validation accuracies reach about their maximum value of 1 (or 100%), as illustrated in Figure 5 and Figure 6, this does not guarantee high performance in predicting the test data, which has not been included in the model’s training at all.

It is evident from Figure 5 and Figure 6 that some of the ANN models do not achieve maximum accuracy on the training and validation data during training. However, while some models do attain an accuracy of about 1 (or 100%), this does not guarantee that these models can correctly predict all labels of new, unseen data. Therefore, despite comparing the training accuracy diagrams in Figure 5 and Figure 6, we selected the best hyperparameters based on the models’ performance on the test data. As discussed, the models were evaluated using a test dataset that included 658 files.

In the first step, we modified the sample rate values during the preprocessing of the acoustic signals. After training three models with sample rates of 4500, 9000, and 18,000 Hz, respectively, we tested these models using the test data. The results are summarized in Table 4, which shows that the model with the lowest sample rate yielded the best results concerning the micro average, macro average, weighted average, and samples average. This model achieved 100%, 77%, 87% for the precision, recall, and F1 score, respectively. However, this model could not predict the labels 8, 10, and 11 well and some the prediction scores fell down to less than 10%. This shows that this model needs to be more investigated to increase its performance. The two other models trained with the data extracted with 9000 and 18,000 Hz showed a lower performance in comparison to the first model by 1 to 7% in the average scores. However, all the models exhibited poor scores for some labels (even below 10%), particularly in recall and F1 score, indicating the need for improvement through other hyperparameters. At first glance, it may seem that gathering more features from each signal by applying a higher sample rate would result in richer training data, leading to better training and prediction. However, the results showed the opposite trend. The reason for these results may be that the acoustic data are not very complex and training its behavior into the ANN models does not require highly extracted features. Overall, a sample rate of 4500 was chosen as the best sample rate for further testing.

In the next step, the length of the Fast Fourier Transform window (n_fft) was considered with values of 256, 512, and 1024, and the prediction scores were compared to identify the best model based on these scores. Among the three tested models, the one with n_fft equal to 1024 achieved the highest scores across micro average, macro average, weighted average, and sample average on the test data, as shown in Table 5. Notably, this model maintained an average score of over 80% in all except the macro average of the recall which dropped to 78%. The two other models which had been trained with 256 and 512 n_fft resulted in poor results by up to 9% lower average scores in comparison to the model which had been trained with 1024 n_fft. All the three models had poor results in predicting the labels 8, 10, and 11. The model which used the 256 n_fft predicted the label 8 by 9% and 16% for recall and F1 score. This model also predicted the label 10 by 6% and 12% for recall and F1 score and label 11 by 14 and 25% for recall and F1 score. Moreover, the model which used the 512 n_fft predicted the label 8, 10, and 11 by 4% to 20% for recall and 7% to 34% for F1 score. However, the model which used the 1027 n_fft predicted the label 8, 10, and 11 by 10% to 64% for recall and 18% to 78% for F1 score.

The signal segments are transformed from time domain to frequency domain by the Fast Fourier Transform and the wider segment has more frequency information available. These results indicate that when capturing a segment of the signal to extract its features, increasing the length of the capturing window can lead to the extraction of more useful information from the signal for training the ANN model. This suggests that capturing more details with wider windows enhances feature extraction, providing better training data for each acoustic signal. Overall, although the model with n_fft equal to 1024 resulted in high prediction scores, there are still recall and F1 scores (for label 10) falling below 20%, which is unacceptable. Therefore, we aimed to improve these scores and investigate the effect of hop length on them in the next stage.

Changing the hop length from 128 to 512 did not significantly affect the average scores, as observed in the results presented in Table 6. However, increasing the hop length from 128 to 256 led to an improvement of 1% in some of the average recall and F1 scores. As shown in Table 6, after changing the hop length from 128 to 256, average precision, recall, and F1 scores changed from 100%, 83%, and 91% to 100%, 84%, and 91% for the micro average scores, respectively, and such a similar increase was observed in the other average scores. The low prediction scores can be observed again in the prediction of some labels like label 10 which has been predicted by the first model by 100%, 10%, and 18% for the precision, recall and, F1, respectively. This label has been predicted by the second model by 100%, 11%, and 20% for the precision, recall, and F1, respectively. The third model also had poor results on the label 10 by 100%, 6%, and 12% for the precision, recall, and F1, respectively. These results showed that we have not arrived at an overall acceptable performance yet and the investigation should be continued to improve the prediction results.

The limited impact of different hop length values may be attributed to the fact that the hop length defines the number of samples between successive frames. Changing the hop length only changes the relative positions between the consecutive frames and the frames might become closer to or further from each other. When they are closer to each other, the common part between them is further apart. This means that when using smaller hop lengths, there is more information that is extracted by both consecutive frames. Because the AI model does not need to consider this information twice, it might seem that using higher hop lengths (resulting in less repetitive extracted data) could be preferable, but as our results show, setting the hop length in a very high value (512) has decreased the recall and F1 score from 84% and 91% to 77% and 87%, respectively. This negative effect is in relation with the windowing function effect. If the windowing function has zero or near zero values at its start and end part, when applying it to the segment, it will lead to a loss of some of the signal information. To address this challenge, using an overlap between each two consecutive frames can decrease this effect. So, when very high values of hop length are applied between two consecutive frames, the common part between them is decreased and the negative effect of the windowing function may happen, resulting in the loss of some important information, which may affect the trained model’s performance. Considering both the positive and negative aspects of applying the high and low values of hop length, selecting an optimum value is suggested, which, in our case, was 256.

Increasing the number of MFCC from 20 to 40 resulted in a significant improvement in recall and F1 score, particularly for label 10, which had been problematic for all previous models. The recall and F1 score for label 10 increased from 11% and 20% to 41% and 58%, respectively. Additionally, increasing the number of MFCC from 20 to 40 led to approximately a 2% improvement in average recall and F1 scores; however, the average precision declined by 1% to 2% across micro, macro, and weighted averages. As depicted in Table 7, the second model (with 40 MFCC) obtained 99%, 86%, and 92% for the precision, recall, and F1, respectively, considering the micro average scores. The rest of the model’s scores were approximately close to the micro average scores with a 5% tolerance.

Remarkably, increasing the number of MFCC from 40 to 80 exceeded expectations, boosting the recall and F1 score for the label 10 from 41% and 58% to an impressive 100% for both metrics. This model, with 80 MFCC, also achieved 100% in all average scores, which is quite remarkable. When we look at the prediction scores for all the labels, we can see that the model has predicted them by obtaining the highest maximum precision, recall, and F1 score, which is 100%, and there are only some 99% scores for labels 3, 9, and 12. As described, the final result of obtaining MFCC from an acoustic signal is a set of coefficients which represent the behavior of the signal. Representing the acoustic signal behavior could be performed by an arbitrary number of coefficients based on the case study. In our case study, obtaining better results by training the model with larger values of MFCC in preprocessing procedure suggests that using MFCC with more coefficients provides a better representation of the acoustic signal and is more suitable for training the ANN model.

## 4. Conclusions

AI models have found their place in various fields, including manufacturing. However, these models face certain challenges, such as the need for significant time and cost investments to prepare sufficient training data. To address this issue, we propose using acoustic-based data as training data. This type of data are not only easy to record with a simple microphone but also contains valuable information about process behavior. This way, we could solve the problem by gathering enough training data. This method of gathering data does not have high costs in comparison to other methods like the acoustic emission method since it needs only a microphone to receive the generated sounds.

In this study, the hybrid EDT process was considered as a case study. It is a complex process with multiple influencing parameters. However, there were concerns regarding the suitability of these data for training an accurate model. Therefore, we decided to evaluate this capability, especially since the models are expected to predict various labels, such as surface roughness, Tons, currents, and rotational speeds, separately using ANNs for multilabel classification, rather than using typical classification models which had been used in the previous studies. After recording the sounds generated during EDT under different Tons, currents, and rotational speeds, the recorded sounds were used as training data for the ANN models. Since acoustic data needs to be preprocessed and its features extracted before being fed as input into the ANN models, we utilized the Librosa package for preprocessing. Due to the varying parameters involved in preprocessing—such as sample rate, FFT window length, hop length, and the number of MFCC—which can significantly impact the prediction results, we aimed to identify the optimal preprocessing parameters for the current dataset. While many researchers may prefer using the Python packages in default setting, our results proved that it is necessary to delve into preprocessing parameters to find their best values resulting in the highest possible performance for the trained model. The final trained AI model with the highest prediction performance can be used in surface roughness evaluation as an alternative method or it can be used to online monitoring of the EDT process by developing a mobile app which has a microphone to receive the generated acoustic signals. Furthermore, the authors believe that the obtained results can be expanded for other manufacturing processes applying some adjustments in the collected data and the programming codes. Our approach considered maximizing precision, recall, and F1 scores across four steps, and the results are presented as follows:

1. Among the three different sampling rates tested (4500 Hz, 9000 Hz, and 18,000 Hz), applying a sample rate of 4500 Hz resulted in better training outcomes, with all average prediction scores exceeding 69%. This performance surpassed that of the two other ANN models. These results indicate that increasing the rate of data collection from recorded sounds does not necessarily lead to more effective training data.
2. Changing the length of the FFT windows significantly affects the prediction scores. Among the three considered values of n_fft (256, 512, and 1024), the model using the highest n_fft yielded the best prediction scores. All average scores in precision, recall, and F1 score improved by more than 9% compared to the best average scores from the previous models. These results demonstrate that capturing more details with wider windows can enhance the feature extraction of the acoustic signal used for training data.
3. The value of hop length did not significantly affect the training and prediction results. However, increasing the hop length from 128 to 256 led to a 1% increase in some average recall and F1 scores. Therefore, the number of samples between successive frames, ranging from 128 to 512, did not have a remarkable effect on the prediction scores.
4. Increasing the number of MFCC in preprocessing significantly affected the prediction results. While increasing this parameter from 20 to 40 improved the average recall and F1 scores by only 2%, raising it from 40 to 80 led to a substantial enhancement in the prediction scores. The corresponding ANN model achieved maximum average scores of 100%. This model demonstrated exemplary performance, even when predicting labels such as label 10, for which the best recall and F1 scores in all previously trained models were only 41% and 58%, respectively.

## Figures and Tables

**Table 1:** Microphone specifications.

Sensitivity	Gain Range	Frequency	S/N	Max SPL	Working Temperature	Storage Temperature
−35 dB ± 2 dB	Maximum +10 dB	60 Hz–18 kHz	>65 dB	100 dB	0~+50 °C	−20~+60 °C

**Table 2:** Tests and the experimental details.

Test No	Rotational Speed (rpm)	Ton (µs)	Amperage (A)	Surface Roughness (µm)
1	10	100	2.25	2.26
2	10	100	1.5	2.22
3	10	200	2.25	2.35
4	10	200	1.5	2.29
5	20	100	2.25	1.86
6	20	100	1.5	1.86
7	20	200	2.25	2.18
8	20	200	1.5	1.97

**Table 3:** The optimum hyper-parameters for the model structure.

Hyper-Parameters	Range	Optimum Hyper-Parameters
Number of Layers	1 to 4	4
Number of Neurons	4 to 64	64-48-44-24
Learning Rate	0.001 to 0.1	0.01

**Table 4:** Classification report and the scores for 4500, 9000, and 18,000 Hz sample rates.

**Sample Rate: 4500 Hz**				
	**Precision**	**Recall**	**F1-Score**	**Support**
0	1.00	0.88	0.93	333
1	1.00	0.93	0.96	325
2	0.99	0.86	0.92	331
3	1.00	0.86	0.92	327
4	1.00	0.75	0.85	331
5	1.00	0.72	0.84	327
6	1.00	1.00	1.00	86
7	0.98	0.81	0.89	80
8	1.00	0.09	0.16	81
9	1.00	0.76	0.86	86
10	1.00	0.06	0.12	81
11	1.00	0.14	0.25	84
12	1.00	0.87	0.93	79
13	1.00	1.00	1.00	81
micro avg	1.00	0.77	0.87	2632
macro avg	1.00	0.69	0.76	2632
weighted avg	1.00	0.77	0.84	2632
samples avg	0.96	0.77	0.83	2632
**Sample Rate: 9000 Hz**				
	**Precision**	**Recall**	**F1-Score**	**Support**
0	1.00	0.92	0.96	333
1	0.99	0.96	0.98	325
2	1.00	0.83	0.91	331
3	1.00	0.91	0.95	327
4	1.00	0.74	0.85	331
5	1.00	0.52	0.68	327
6	1.00	1.00	1.00	86
7	0.99	0.86	0.92	80
8	1.00	0.01	0.02	81
9	1.00	0.90	0.94	86
10	1.00	0.05	0.09	81
11	0.00	0.00	0.00	84
12	1.00	0.75	0.86	79
13	1.00	0.99	0.99	81
micro avg	1.00	0.75	0.86	2632
macro avg	0.93	0.67	0.73	2632
weighted avg	0.97	0.75	0.82	2632
samples avg	0.97	0.75	0.82	2632
**Sample Rate: 18,000 Hz**				
	**Precision**	**Recall**	**F1-Score**	**Support**
0	1.00	0.98	0.99	333
1	1.00	0.90	0.95	325
2	1.00	0.77	0.87	331
3	0.99	0.83	0.91	327
4	1.00	0.68	0.81	331
5	0.99	0.58	0.73	327
6	1.00	1.00	1.00	86
7	1.00	0.80	0.89	80
8	1.00	0.43	0.60	81
9	1.00	0.67	0.81	86
10	1.00	0.01	0.02	81
11	1.00	0.05	0.09	84
12	0.99	0.90	0.94	79
13	0.96	1.00	0.98	81
micro avg	0.99	0.75	0.85	2632
macro avg	0.99	0.69	0.76	2632
weighted avg	0.99	0.75	0.82	2632
samples avg	0.98	0.75	0.81	2632

**Table 5:** Classification report and the scores for 256, 512, and 1024 n_fft.

**n_fft: 256**				
	**Precision**	**Recall**	**F1-Score**	**Support**
0	1.00	0.88	0.93	333
1	1.00	0.93	0.96	325
2	0.99	0.86	0.92	331
3	1.00	0.86	0.92	327
4	1.00	0.75	0.85	331
5	1.00	0.72	0.84	327
6	1.00	1.00	1.00	86
7	0.98	0.81	0.89	80
8	1.00	0.09	0.16	81
9	1.00	0.76	0.86	86
10	1.00	0.06	0.12	81
11	1.00	0.14	0.25	84
12	1.00	0.87	0.93	79
13	1.00	1.00	1.00	81
micro avg	1.00	0.77	0.87	2632
macro avg	1.00	0.69	0.76	2632
weighted avg	1.00	0.77	0.84	2632
samples avg	0.96	0.77	0.83	2632
**n_fft: 512**				
	**Precision**	**Recall**	**F1-Score**	**Support**
0	1.00	0.87	0.93	333
1	1.00	0.84	0.91	325
2	1.00	0.76	0.87	331
3	1.00	0.85	0.92	327
4	1.00	0.80	0.89	331
5	1.00	0.72	0.83	327
6	1.00	1.00	1.00	86
7	1.00	0.79	0.88	80
8	1.00	0.14	0.24	81
9	0.99	0.92	0.95	86
10	1.00	0.04	0.07	81
11	1.00	0.20	0.34	84
12	1.00	0.92	0.96	79
13	1.00	0.99	0.99	81
micro avg	1.00	0.76	0.86	2632
macro avg	1.00	0.70	0.77	2632
weighted avg	1.00	0.76	0.84	2632
samples avg	0.92	0.76	0.81	2632
**n_fft: 1024**				
	**Precision**	**Recall**	**F1-Score**	**Support**
0	1.00	0.91	0.95	333
1	1.00	0.94	0.97	325
2	1.00	0.95	0.97	331
3	1.00	0.89	0.94	327
4	1.00	0.84	0.91	331
5	1.00	0.66	0.80	327
6	1.00	1.00	1.00	86
7	1.00	0.94	0.97	80
8	0.98	0.52	0.68	81
9	1.00	0.80	0.89	86
10	1.00	0.10	0.18	81
11	1.00	0.64	0.78	84
12	1.00	0.77	0.87	79
13	1.00	0.98	0.99	81
micro avg	1.00	0.83	0.91	2632
macro avg	1.00	0.78	0.85	2632
weighted avg	1.00	0.83	0.89	2632
samples avg	0.96	0.83	0.87	2632

**Table 6:** Classification report and the scores for 128, 256, and 512 hop length.

**Hop Length: 128**				
	**Precision**	**Recall**	**F1-Score**	**Support**
0	1.00	0.91	0.95	333
1	1.00	0.94	0.97	325
2	1.00	0.95	0.97	331
3	1.00	0.89	0.94	327
4	1.00	0.84	0.91	331
5	1.00	0.66	0.80	327
6	1.00	1.00	1.00	86
7	1.00	0.94	0.97	80
8	0.98	0.52	0.68	81
9	1.00	0.80	0.89	86
10	1.00	0.10	0.18	81
11	1.00	0.64	0.78	84
12	1.00	0.77	0.87	79
13	1.00	0.98	0.99	81
micro avg	1.00	0.83	0.91	2632
macro avg	1.00	0.78	0.85	2632
weighted avg	1.00	0.83	0.89	2632
samples avg	0.96	0.83	0.87	2632
**Hop Length: 256**				
	**Precision**	**Recall**	**F1-Score**	**Support**
0	1.00	0.92	0.96	333
1	1.00	0.92	0.96	325
2	1.00	0.91	0.95	331
3	1.00	0.94	0.97	327
4	1.00	0.82	0.90	331
5	1.00	0.76	0.86	327
6	1.00	1.00	1.00	86
7	1.00	0.84	0.91	80
8	1.00	0.57	0.72	81
9	0.99	0.87	0.93	86
10	1.00	0.11	0.20	81
11	1.00	0.46	0.63	84
12	1.00	0.89	0.94	79
13	1.00	0.99	0.99	81
micro avg	1.00	0.84	0.91	2632
macro avg	1.00	0.79	0.85	2632
weighted avg	1.00	0.84	0.90	2632
samples avg	0.97	0.84	0.88	2632
**Hop Length: 512**				
	**Precision**	**Recall**	**F1-Score**	**Support**
0	1.00	0.85	0.92	333
1	1.00	0.94	0.97	325
2	1.00	0.85	0.92	331
3	1.00	0.81	0.90	327
4	1.00	0.80	0.89	331
5	1.00	0.61	0.75	327
6	1.00	1.00	1.00	86
7	1.00	0.85	0.92	80
8	1.00	0.16	0.28	81
9	1.00	0.79	0.88	86
10	1.00	0.06	0.12	81
11	1.00	0.56	0.72	84
12	1.00	0.72	0.84	79
13	0.98	1.00	0.99	81
micro avg	1.00	0.77	0.87	2632
macro avg	1.00	0.71	.0.79	2632
weighted avg	1.00	0.77	0.85	2632
samples avg	0.94	0.77	0.82	2632

**Table 7:** Classification report and the scores for 20, 40, and 80 MFCC.

**mfcc: 20**				
	**Precision**	**Recall**	**F1-Score**	**Support**
0	1.00	0.92	0.96	333
1	1.00	0.92	0.96	325
2	1.00	0.91	0.95	331
3	1.00	0.94	0.97	327
4	1.00	0.82	0.90	331
5	1.00	0.76	0.86	327
6	1.00	1.00	1.00	86
7	1.00	0.84	0.91	80
8	1.00	0.57	0.72	81
9	0.99	0.87	0.93	86
10	1.00	0.11	0.20	81
11	1.00	0.46	0.63	84
12	1.00	0.89	0.94	79
13	1.00	0.99	0.99	81
micro avg	1.00	0.84	0.91	2632
macro avg	1.00	0.79	0.85	2632
weighted avg	1.00	0.84	0.90	2632
samples avg	0.97	0.84	0.88	2632
**mfcc: 40**				
	**Precision**	**Recall**	**F1-Score**	**Support**
0	1.00	1.00	1.00	333
1	1.00	0.98	0.99	325
2	1.00	0.90	0.95	331
3	0.99	0.99	0.99	327
4	1.00	0.60	0.75	331
5	0.95	0.88	0.91	327
6	1.00	1.00	1.00	86
7	1.00	0.81	0.90	80
8	0.92	0.98	0.95	81
9	0.97	0.45	0.62	86
10	1.00	0.41	0.58	81
11	1.00	0.52	0.69	84
12	0.93	1.00	0.96	79
13	1.00	0.81	0.90	81
micro avg	0.99	0.86	0.92	2632
macro avg	0.98	0.81	0.87	2632
weighted avg	0.99	0.86	0.90	2632
samples avg	0.98	0.86	0.89	2632
**mfcc: 80**				
	**Precision**	**Recall**	**F1-Score**	**Support**
0	1.00	1.00	1.00	333
1	1.00	1.00	1.00	325
2	1.00	1.00	1.00	331
3	1.00	0.99	1.00	327
4	1.00	1.00	1.00	331
5	1.00	1.00	1.00	327
6	1.00	1.00	1.00	86
7	1.00	1.00	1.00	80
8	1.00	1.00	1.00	81
9	1.00	0.99	0.99	86
10	1.00	1.00	1.00	81
11	1.00	1.00	1.00	84
12	1.00	0.99	0.99	79
13	1.00	1.00	1.00	81
micro avg	1.00	1.00	1.00	2632
macro avg	1.00	1.00	1.00	2632
weighted avg	1.00	1.00	1.00	2632
samples avg	1.00	1.00	1.00	2632

## Data Availability

Data are unavailable due to privacy restrictions.
